# A Novel Single-Site Mutation in the Catalytic Domain of Protoporphyrinogen Oxidase IX (PPO) Confers Resistance to PPO-Inhibiting Herbicides

**DOI:** 10.3389/fpls.2019.00568

**Published:** 2019-05-15

**Authors:** Gulab Rangani, Reiofeli A. Salas-Perez, Raphael A. Aponte, Michael Knapp, Ian R. Craig, Thomas Mietzner, Ana Claudia Langaro, Matheus M. Noguera, Aimone Porri, Nilda Roma-Burgos

**Affiliations:** ^1^Department of Crop, Soil, and Environmental Sciences, University of Arkansas, Fayetteville, AR, United States; ^2^BASF SE, Ludwigshafen, Germany; ^3^Department of Crop Science, Universidade Federal Rural do Rio de Janeiro, Seropédica, Brazil

**Keywords:** **Amaranthus palmeri**, computational modeling, cross-resistance, Palmer amaranth, PPO inhibitors, PPO mutation, protoporphyrinogen oxidase, target-site resistance

## Abstract

Protoporphyrinogen oxidase (PPO)-inhibiting herbicides are used to control weeds in a variety of crops. These herbicides inhibit heme and photosynthesis in plants. PPO-inhibiting herbicides are used to control *Amaranthus palmeri* (Palmer amaranth) especially those with resistance to glyphosate and acetolactate synthase (ALS) inhibiting herbicides. While investigating the basis of high fomesafen-resistance in *A. palmeri*, we identified a new amino acid substitution of glycine to alanine in the catalytic domain of PPO2 at position 399 (G399A) (numbered according to the protein sequence of *A. palmeri*). G399 is highly conserved in the PPO protein family across eukaryotic species. Through combined molecular, computational, and biochemical approaches, we established that PPO2 with G399A mutation has reduced affinity for several PPO-inhibiting herbicides, possibly due to steric hindrance induced by the mutation. This is the first report of a PPO2 amino acid substitution at G399 position in a field-selected weed population of *A. palmeri*. The mutant *A. palmeri* PPO2 showed high-level *in vitro* resistance to different PPO inhibitors relative to the wild type. The G399A mutation is very likely to confer resistance to other weed species under selection imposed by the extensive agricultural use of PPO-inhibiting herbicides.

## Introduction

Weeds have been the major cause of crop yield losses since the dawn of agriculture. Herbicides are the primary tools used to manage weedy species. Weeds continue to be a growing threat to food security because of resistance to herbicides (Heap, 2018). The use of herbicides (synthetic organic chemicals) has been the dominant weed management technique since the ‘60s (Délye et al., 2013). While herbicides have greatly improved agricultural production, their intensive, and continuous use has resulted in the evolution of resistance in many weedy plant species (Powles and Yu, 2010). Currently, there are >480 unique cases of herbicide resistance reported globally, distributed over 252 weedy species and encompassing resistance to almost all (27) herbicide mechanisms of action (Heap, 2018).

Protoporphyrinogen IX oxidase (PPO) is one of the potent herbicide targets (Hao et al., 2011). It is a highly conserved family of membrane-associated proteins in both eukaryotes and prokaryotes (Arnould et al., 1999). PPO catalyzes the oxidation of protoporphyrinogen IX to protoporphyrin IX, the last common intermediate for the tetrapyrrole synthesis system, which exists universally in all heme and chlorophyll-synthesizing organisms (Deybach et al., 1985; Dayan and Dayan, 2011). Upon treatment with PPO-herbicides, susceptible plants accumulate protoporphyrinogen IX due to inhibition of PPO. Protoporphyrinogen IX is transported to the cytoplasm, where its reaction with unspecific plasma membrane-bound peroxidase spontaneously form protoporphyrin IX. Protoporphyrin IX accumulates in the cytoplasm and, in the presence of light, generates singlet oxygen that causes lipid peroxidation. In other words, it causes degradation of cell membranes and cell lysis (Matringe and Scalla, 1988; Becerril and Duke, 1989; Sandmann et al., 1990; Jacobs et al., 1991; Jacobs and Jacobs, 1993; Lee and Duke, 1994). Several classes of PPO-inhibiting herbicides such as diphenylethers, thiadiazoles, oxadiazoles, triazolinones, N-phenyl-phthalimides, and pyrimidinediones inhibit the PPO enzyme.

In plants, two nuclear *PPO* genes encode organelle-specific isoforms of PPO, namely, PPO1 (plastid-targeted, where chlorophyll and heme are synthesized) and PPO2 (mitochondria-targeted, where heme is produced) (Lermontova et al., 1997). In some plant species, PPO2 is dual-targeted to mitochondria and chloroplast (Watanabe et al., 2001; Patzoldt et al., 2006). PPO converts protoporphyrinogen IX into protoporphyrin IX in both organelles; thus, both PPO1 and PPO2 are targets for PPO-inhibiting herbicides. PPO-inhibiting herbicides became widely used during 1980–1990. However, after the introduction of glyphosate-resistant crops in the mid-1990s, usage of PPO inhibitors and other herbicides declined until 2006 (USDA-NASS, 2019) when economically damaging weeds evolved resistance to glyphosate. Widespread weed resistance to acetolactate synthase (ALS) inhibitors, glyphosate, and various other herbicides used in crop production revived high usage of PPO-inhibiting herbicides recently (Salas et al., 2016; Salas-Perez et al., 2017; Dayan et al., 2018).

Some weed species have evolved resistance to PPO-inhibiting herbicides and this number is increasing gradually. To date, there are 13 PPO-inhibitor-resistant weed species including *A. palmeri* (Heap, 2018). *A. palmeri* has become one of the most troublesome weeds in corn, soybean, cotton, sorghum, and many other crops in the United States (Beckie, 2006; Smith and Scott, 2010; Webster and Nichols, 2012; Ward et al., 2013; Norsworthy et al., 2014; Chahal et al., 2015). Studying the molecular mechanisms of evolved PPO-herbicide resistance in weedy plant species is important and necessary. One utility is to enable rational identification and design of PPO inhibitors that retain strong binding affinity to the various mutated PPO proteins. Metabolic mechanisms have been predicted for the resistance to PPO inhibitors (Trezzi et al., 2009; Dayan et al., 2014; Mangin et al., 2016) because differential herbicide metabolism is the basis for tolerance to these herbicides in several species. However, the field-evolved resistance is generally attributed to target-site mutations thus far (Patzoldt et al., 2006; Rousonelos et al., 2012; Wuerffel et al., 2015; Salas-Perez et al., 2017; Varanasi et al., 2018).

Four amino acid mutations at two sites in PPO2 have been selected in resistant weedy species. First is a *PPO2* gene mutation that leads to a deletion of Gly210 (ΔG210) in the PPO2 enzyme of *A*. *tuberculatus* (Patzoldt et al., 2006). This deletion mutation was also found recently in *A. palmeri* (Salas et al., 2016; Salas-Perez et al., 2017). Second is a substitution of Arg98 with leucine (R98L) in *Ambrosia artemisiifolia*. The two most recently identified ones occur at the same position in PPO-resistant *A. palmeri* as R128G or R128M (Patzoldt et al., 2006; Rousonelos et al., 2012; Salas et al., 2016; Giacomini et al., 2017; Salas-Perez et al., 2017; Varanasi et al., 2018). The R128 locus is the same as R98; the numbering change is due to the presence of a 30-amino acid signal peptide in *A. palmeri*.

In 2016, we reported resistance to fomesafen and other PPO inhibitors in *A*. *palmeri* in Arkansas, United States. The presence of target-site mutations ΔG210 and R128G conferred high to moderate level of resistance in many of the field-evolved resistant populations (Salas et al., 2016; Salas-Perez et al., 2017). In the present study, we identified a new substitution mutation in the catalytic domain of the PPO2 enzyme, which also endows broad resistance to PPO inhibitors in *A*. *palmeri*. Our data indicate that the resulting mutation in PPO2, glycine399 to alanine, alters the herbicide-binding site, which reduces sensitivity to various PPO-inhibiting herbicides, as observed in field-selected plants.

## Materials and Methods

### Plant Material, Growth Conditions, and Herbicide Applications

The resistant Palmer amaranth population designated as MIS-D was used in this study. MIS-D field population was confirmed resistant to glyphosate, PPO- and ALS-inhibiting herbicides (Salas-Perez et al., 2017). The susceptible biotype (SS) was collected in Crawford County, Arkansas, and is susceptible to all herbicides tested in previous experiments (Salas et al., 2016). Plants from MIS-D field population that survived the recommended dose of fomesafen (264 g ai ha^-1^) were used for sequencing the *PPO2* gene. Four plants (two male and two female), were confirmed by gene sequencing to contain the mutation leading to alanine in 399 position (G399A), and were isolated to generate F1 populations. Two male and two female *A. palmeri* plants that were heterozygous and homozygous for the G399A mutation, respectively, were allowed to hybridize to produce R_39_P and R_43_P F1 populations, respectively. Plants were grown in the greenhouse maintained at 32/25 ± 3°C day/night temperature with supplemental light for 14 h. Seeds from SS, MIS-D (original field population), R_39_P, and R_43_P populations were used for bioassays in the greenhouse. Herbicide treatments were applied with a laboratory sprayer equipped with a 110° flat-fan spray nozzle (Teejet; Spraying Systems Co., Wheaton, IL, United States) delivering 187 L ha^-1^ of herbicide solution at 310 kPa. The nozzle was set at 51 cm above the plant canopy. Plants were returned to the greenhouse immediately after herbicide treatment. The herbicides were applied when the plants were 5- to 7-cm tall.

### cDNA Sequencing

Some plants from MIS-D in the bioassay experiment were assigned ID numbers and ∼25 mg of leaf tissues were collected before spraying fomesafen. After evaluation of plant response, the leaf tissues collected from survivors were labeled as R while those from plants, which did not survive the herbicide application were labeled as S. Total RNA was isolated using the Plant RNeasy Mini Kit (Qiagen), treated with DNAse (Thermo Fisher Scientific), and converted to cDNA using Access RT-PCR System (Promega). Full-length *PPO2* were amplified using 1F (5′-GG GGTACCCGGGTAAACTGATCTTATGTTAATTC-3′) and 3R (5′- GGAATTCGAGCTCGCATGCTTACGCGGTCTTCTCAT CATC-3′) primer pair, purified, and sent for sequencing using internal primers. The PCR was conducted in a 40-μL volume that consisted of 2 μL (60 ng) of cDNA, 0.5 μM of each primer, and 20 μL of EmeraldAmp MAX PCR Master Mix (Takara). The PCR was run with the following profile: 98°C for 30 s; 40 cycles of 98°C for 1 min, 58°C for 1 min and 72°C for 2 min; followed by a final extension step for 10 min at 72°C. Overlapping internal primers used for sequencing are as follows: Apx2-1F (5′- GTAATTCAATCCATTAC CCAC CTT 3′), Apx2-1R (5′-TTCCATA CGTCGGGAAATGT-3′), Apx2-2F (5′-TGTTGGAACCATTTCTCTGG-3′), Apx2-2R (5′-GGGGATAAGAACTCCGA AGC-3′), Apx2-3F (5′-GATG CTGTGGTTGTCACTGC-3′), and Apx2-3R (5′-TTACGCGG TCTTCTCATCCAT-3′). The PCR product purified from agarose gel using Pure-Link Quick Gel Extraction Kit (Invitrogen) was sent to Eurofins Genomics for sequencing. The resulting overlapping fragments from all individual R and S plants were assembled into one sequence using Sequencher 5.4.6 (Gene Codes Corporation) to obtain the complete coding region of the *PPO2* gene. The nucleotide sequences were translated into open reading frames using the online ExPASy translation tool.

### Computational Modeling

Two structural models of *A. palmeri* PPO2 protein, S-PPO2 and R-PPO2, were built using the homology modeling workflow in YASARA (YASARA, version 17.4; YASARA Biosciences GmbH, Vienna, Austria) using protein data bank (PDB) entry 1SEZ of tobacco PPO2 as template. The S-PPO2 model used a modified version of the resistant PPO sequence in which position 399 was changed back to the wild-type glycine (G399 model). All other sequence positions were as found in the resistant sequence. The R-PPO2 model used the unmodified resistant PPO sequence (A399 model). Default settings and standard protein preparation steps were used. Fomesafen was initially modeled into the binding-site of the wild type S-PPO2 using the docking scripts and tools of YASARA. To gain insight into the effect of the G399A on fomesafen binding, the predicted binding model was then transferred into the R-PPO2 model, where G399 was changed to A399. The changes in protein-ligand interactions due to the G399A mutation were then examined and interpreted.

### Cleaved Amplified Polymorphic Sequences (CAPS) Assay Development and Genotyping

Based on the *PPO2* sequence information obtained from the S and R plants, we developed a CAPS marker for detecting the mutation at 399 amino acid position. We found the mutation from wild type codon GGT to GCT at 399 position. *Rsa*I restriction endonuclease recognizes the sequence GTˆAC, so a G to C mutation eliminates the restriction site. Genomic DNA was extracted from all R plants along with a few S plants and a 691-bp fragment was amplified using the primers, *Rsa*IF (5′-CGTACCCCTTTCCGTTATGA-3′) and *Rsa*IR (5′-CCGGGAAGATCTT TTTCCAT-3′). The amplicon was then subjected to *Rsa*I digestion. Upon digestion, the wild-type amplicon would generate a 505-bp and 185-bp band, whereas the homozygous mutant amplicon would generate the intact 691-bp band (Supplementary Figure S3). A heterozygous plant for the G399A mutation would produce three bands; 691-, 505-, and 185 bp (Supplementary Figure S3). PCR conditions were similar to those described earlier with 30 cycles. Restriction digestions were carried out according to the manufacturer’s recommendations (New England Biolabs), and digestion patterns were viewed on 3% agarose gels with GelRed (electrophoresis at 90–100 V for 70–80 min). Thirty-five fomesafen-resistant Palmer amaranth accessions from a previous study (Salas-Perez et al., 2017) were genotyped to detect the presence of G399A mutation by CAPS assay.

### Fomesafen Dose-Response Assay

Seeds from Palmer amaranth populations, MIS-D, R_39_P, R_43_P, and SS were sown in 15-cm pots filled with commercial potting soil (Sunshine Premix No. 1; Sun Gro Horticulture, Bellevue, WA, United States). Four days after planting, seedlings were thinned to five per pot. When seedlings were 5-7-cm tall, fomesafen (Flexstar, Syngenta, Greensboro, NC, United States) was applied at 16.5 (0.062X), 33 (0.125X), 66 (0.25X), 132 (0.5X), 197.5 (0.75X), 263 (1X), 527 (2X), 1053 (4X), and 2107 (8X) g ai ha^-1^, where 1X represents the recommended dose. The SS population was sprayed with nine doses as well: 1, 2, 4, 8, 16.5, 33, 66, 132, and 263 g ai ha^-1^, corresponding to a range of 1/256 to 1X of the recommended dose. Herbicide treatments included a non-ionic surfactant (Induce, Helena Chemical, Collierville, TN, United States) at 0.5% v/v. A non-treated control was included with each population. The overall effects of fomesafen (stunting, chlorosis, necrosis, and desiccation) were assessed visually on a scale of 0 (no control) to 100% (complete kill) relative to the non-treated control at 21 days after treatment (DAT). The shoot biomass was harvested, dried at 60°C for 72 h, and weighed. Data were analyzed by fitting a non-linear, three-parameter log-logistic regression model using the “drc” package in R 3.5.1 (Ritz et al., 2015). The model used is defined by: Y = d/[1+exp{b[log(x) – log(ED50)]}], where Y is the response (biomass or herbicide injury), d is the asymptote at upper limit, *x* is the fomesafen dose, and *b* is the slope around “ED50,” which is the value of x giving a 50% response of Y. The dose needed to cause 50% herbicide injury (ED_50_) was calculated. The resistance factor was determined by comparing ED_50_ values between the R and SS accessions.

### Cross-Resistance to Foliar-Applied PPO Herbicides

The MIS-D population is cross-resistant to the 1X dose of various foliar-applied PPO herbicides (Salas-Perez et al., 2017). In this follow-up research, the cross-resistance profile of F1 population, R_39_P, was investigated using twice the recommended dose of each PPO herbicide. Seeds from R_39_P and SS populations were planted in cellular trays in the greenhouse. Herbicides representing each PPO herbicide family were applied to 5-cm tall seedlings. Twenty-five seedlings from each population were treated with acifluorfen (1120 g ai ha^-1^), carfentrazone (560 g ai ha^-1^), flumioxazin (141 g ai ha^-1^), fomesafen (528 g ai ha^-1^), lactofen (448 g ai ha^-1^), and saflufenacil (49 g ai ha^-1^). The herbicides were applied with the respective recommended adjuvants. Carfentrazone and flumioxazin treatments included 0.25% non-ionic surfactant (NIS) (v/v), whereas acifluorfen included 0.125% NIS. Lactofen was applied with 0.5% crop oil concentrate (v/v), whereas saflufenacil was sprayed with 1% methylated seed oil, and 1% ammonium sulfate (w/v). Herbicides were applied in a laboratory spray chamber fitted with 800067 flat fan nozzles calibrated to deliver 187 L ha^-1^ at 269 kPa. At 21 DAT, injury, and mortality were evaluated visually.

### Recombinant Expression, Purification, and *in vitro* Inhibition Studies of PPO2

Effects of G399A substitution were studied using *A. tuberculatus* backbone. The glycine at 399 position in *A. palmeri* PPO2 corresponds to G398 in *A. tuberculatus* PPO2, but the said mutation is referred as G399A in this experiment (although *A. tuberculatus* PPO2 backbone was used) to avoid confusion. Recombinant expressions of five other possible G399 substitutions (G399D, G399V, G399C, G399R, and G399S) based on a single nucleotide base change at position one or two of the G399 codon were also investigated. In addition, *in vitro* inhibition of the wild type and previously known PPO2 mutations conferring PPO resistance (ΔG210, R128L) were studied. The *PPO2* gene from *A. tuberculatus* was synthesized *de novo* and subcloned into pRSetB plasmid (Invitrogen, CA, United States) using *Bam*HI and *Hin*dIII restriction sites. The construct contained an N-terminal hexa-histidine tag to facilitate protein purification. The plasmid construct was transformed into *E. coli* strain BL21(DE3)pLysS (Novagen, EMD Millipore Corp., MA, United States) and was selected in LB agar plates supplemented with 100 μg mL^-1^ ampicillin and 34 μg mL^-1^ chloramphenicol. For pre-cultures, 3 mL of LB medium containing ampicillin and chloramphenicol was inoculated using a single colony picked directly from the agar plates, and then cultivated at 37°C with shaking at 200 rpm for 6 h (pre-culture). From this culture, 20 μL was inoculated into a 100-mL flask containing 20 mL LB medium and the same antibiotics. The culture was incubated overnight in a shaker (200 rpm, 37°C). An aliquot (100 μL) of the overnight culture was transferred into a 250-mL flask containing 100 mL ZYM-5052 autoinduction medium (preparation procedure is described in Supplemental Data) with ampicillin (100 μg mL^-1^) and chloramphenicol (34 μg mL^-1^). The main culture was incubated at 37°C for 5 h with shaking at 200 rpm, then for an additional 21 h at 25°C with shaking.

At the end of cultivation, the cells were harvested by centrifugation at 6000 x *g* for 30 min at 4°C. For every gram of pellet, 10 mL of PPO lysis buffer (50 mM NaH_2_PO_4_, 100 mM NaCl, 5 mM imidazole, 5% glycerol (v/v), pH 7.5, 20 mg mL^-1^ lysozyme, 30 unit mL^-1^ DNase I) containing protease inhibitors (complete EDTA free, Roche Diagnostics, Mannheim, Germany) was added. The suspension was sonicated (3 min, 90% amplitude, with 30 s rest intervals) in an ice-bath to avoid overheating the samples. The cell debris was removed by centrifugation at 38,000 × *g* for 30 min at 4°C and the supernatant was added with 2 mL of 5 M NaCl. To purify the enzyme, a 500-μL-bed volume of HisPur Ni-NTA resin (Thermo Fisher Scientific, Illinois, United States) was placed in a column and equilibrated with a buffer (20 mM NaH_2_PO_4_, 50 mM NaCl, 5 mM imidazole, 5 mM MgCl_2_, 17% glycerol (v/v), 0.1 mM EDTA, pH 8.0). The supernatant, which contained the soluble recombinant protein, was passed through the resin. Non-specifically bound proteins were eluted using 5.6-mL wash buffer (20 mM NaH_2_PO_4_, 50 mM NaCl, 5 mM imidazole, 17% glycerol (v/v), adjusted to pH 7.5) and the target protein was eluted in 1 mL of elution buffer [20 mM NaH_2_PO_4_, 50 mM NaCl, 250 mM imidazole, 17% glycerol (v/v), pH 7.5]. The purified enzyme was quantified using Scandrop nano-volume spectrophotometer (Analytikjena, Life Science, Germany). The protein and the cell debris (2.5 μg) was electrophoresed using 10% sodium dodecyl sulfate-polyacrylamide gel electrophoresis to check protein quality and solubility.

Protoporphyrinogen IX oxidase was assayed using the fluorescence assay with excitation and emission wavelength of 405- and 630 nm, respectively. The assay was conducted in a 187-μL reaction mixture containing100 mM Tris–HCl, 1 mM EDTA, 5 mM DTT, 0.0085% Tween 80, and 15 μL of enzyme in resuspension buffer (0.05 M Tris–HCl, pH 7.3, 3.2 mM EDTA, 20% v/v glycerol). The concentration of enzyme differed for each mutant depending on the activity of the enzyme in the presence of saturating concentration of substrate and absence of PPO inhibitor. Maximum fluorometric readings were recorded for the amount of each enzyme. These values were used to calculate the enzyme activity (fluorescence units/minute) compared to the WT. Recombinant protein that showed enzyme activity in the absence of PPO inhibitor was subjected to *in vitro* PPO inhibitor dose-response assay. PPO inhibitors, purchased as Pestanal standards (Sigma-Aldrich, Steinheim, Germany) were dissolved in 80% DMSO. Inhibitors evaluated included diphenylether, pyrimidinedione, triazolinone, N-phenylphthalimide, phenylpyrazole, and thiadiazole herbicides. Ten PPO inhibitor concentrations (10 μL) ranging from 0.00005 M to 5.12 × 10^-12^ M were added to the reaction mixture. The samples were incubated for 30 min at room temperature. Protoporphyrinogen IX (3.24 μM) was then added and the fluorescence was recorded for 30.25 min (33 cycles of 55 s cycle^-1^) using a microplate reader (CLARIOstar, BMG LabTech, Germany). The substrate protoporphyrinogen IX was prepared from protoporphyrin using sodium amalgam reduction (preparation procedure described in Supplemental Data). The slope (per min) was recorded and the percent inhibition was calculated relative to the slope of the positive and negative control. The assay was conducted with two replicates. Inhibitor concentrations (M) required to reduce PPO2 activity by 50% (IC_50_ values) were estimated using non-linear regression procedures (three-parameter or four-parameter log-logistic function). For each inhibitor, resistance factors were calculated by dividing the IC_50_ values of mutant with the sensitive PPO2.

## Results

### A Novel Single Amino Acid Substitution Mutation as the Basis of Fomesafen Resistance in *A. palmeri* Population

A field population, MIS-D, has high resistance to fomesafen (Salas-Perez et al., 2017). In the same study, we found that the majority of survivors did not carry the G210 deletion mutation (ΔG210) that confers resistance to PPO inhibitors (Patzoldt et al., 2006). To determine if another target site mutation is the basis for high-level resistance in this population, the *PPO2* of five resistant (R) plants and three fomesafen-sensitive (S) plants was sequenced using cDNA. The full-length cDNA of 1608 bp codes for the dual targeting signal peptide, the membrane-binding domain, the FAD-binding domain, and the substrate-binding domain of mature mitochondrial PPO2 protein (based on published sequence from *A*. *hypochondriacus*, EU024569, and *A*. *palmeri*, MF583744). The sequences we obtained were 1,608 bp; therefore, we generated full-length cDNA sequences of PPO2 from R and S plants (GenBank accession numbers: MK408971, MK408972, MK408973, MK408974, MK408975, MK408976, MK408977, and MK408978). Comparison of the cDNA-inferred amino acid sequences of the PPO2 protein revealed three amino acid polymorphisms in R plants; however, only a single change in amino acid, glycine to alanine at position 399 (G399A) (numbered relative to the full-length sequence of *A*. *palmeri*, ATE88443), consistently differed between all R and S plants (Supplementary Figure S1). The glycine to alanine substitution resulted from a codon change of GGT to GCT at position 399. Except for G399A, other previously reported resistance-conferring mutations (ΔG210, R128G, or R128M) were not found in R plants.

**FIGURE 1:**
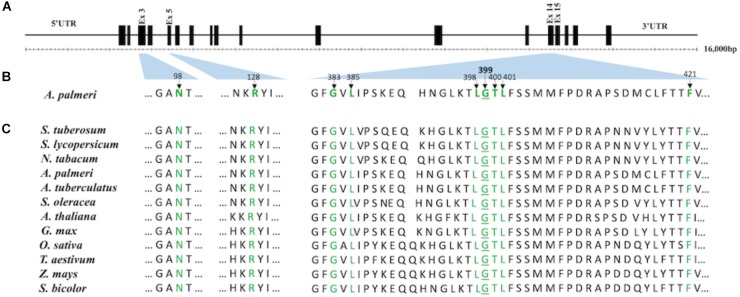
Representation of conserved amino acids in the catalytic domain of PPO2 protein of *A. palmeri* and its corresponding location in the *PPO2* gene **(A)**. Schematic map of the reference *PPO2* gene from *A. hypochondriacus* (EU024569). The gene length and location of exons (black box) are drawn to scale. The gene has 18 predicted exons. Thin lines represent 5′ UTR, introns and 3′ UTR. **(B)** Graphical representation of the positions of conserved amino acids (highlighted in green) in the PPO2 protein of *A*. *palmeri*. The location of corresponding genetic code is shown by the light blue-shaded area between the *PPO2* gene **(A)** and protein sequence **(B)**. The following amino acids are believed to be involved in substrate or inhibitor binding, as deduced from the crystal structure of *N. tabacum* PPO2 protein: Asn67, Arg98, Gly354, Leu356, Leu369, Gly370, Tyr371, Leu372, and Phe392 (Koch *et al*., 2004). Their corresponding loci in *A*. *palmeri* are Asn98, Arg128, Gly383, Leu385, Leu398, Gly399, Tyr400, Leu401, and Phe421. The difference in numbering with respect to *N. tabacum* is due to the presence of 30 amino acid signal peptide extension at the N terminal of PPO2 in *A. palmeri*. Amino acid numbering is based on *A*. *Palmeri* (ATE88443) starting at methionine. **(C)** Alignment of the PPO2 amino acid sequence spanning the catalytic domain among different plant species. PPO2 protein sequences from *Solanum tuberosum* (CAA1240*1), S. lycopersicum* (NP_001335305), *S. oleracea* (BAB60710), *Glycine max* (NP_001236376), *Arabidopsis* (NP_196926*), A. Palmeri* (ATE88443*), A. tuberculatus* (ABD52329), *N. tabacum* (BAA34712), *Oryza sativa* (XP_025880545), *Triticum aestivum* (SPT18865), *Zea mays* (NP_001105004), and *Sorghum bicolor* (XP_002446710) were aligned using CLUSTAL-W (https://www.genome.jp/tools/clustalw).

Using the grain amaranth (*A*. *hypochondriacus*, EU024569) genomic sequence of *PPO2* as reference, we found that the genetic code for G399 resides on exon 15 (Figure 1A). The corresponding change in amino acid (G399A) in R plants is located in the catalytic domain of PPO2 protein (Figure 1B) based on the resolved protein structure of *Nicotiana tabacum* (tobacco) PPO2 (Koch et al., 2004). The G399A mutation is not among the previously reported resistance-conferring mutations from PPO-resistant weeds (Patzoldt et al., 2006; Salas et al., 2016; Giacomini et al., 2017; Salas-Perez et al., 2017). An examination of various eukaryotic PPO proteins revealed that the glycine at position 399 is highly conserved among wild type PPO2 and PPO1 (Figure 1C and Supplementary Figure S2). We found only an alanine mutation at position 399 among the R plants sequenced. As PPO1 is also a binding target for PPO-inhibiting herbicides, we sequenced the *PPO1* gene in the same R and S plants and did not find any polymorphisms among R plants from MIS-D population.

### Molecular Hypothesis for G399A Mutation in PPO2 Using Computational Modeling

**FIGURE 2:**
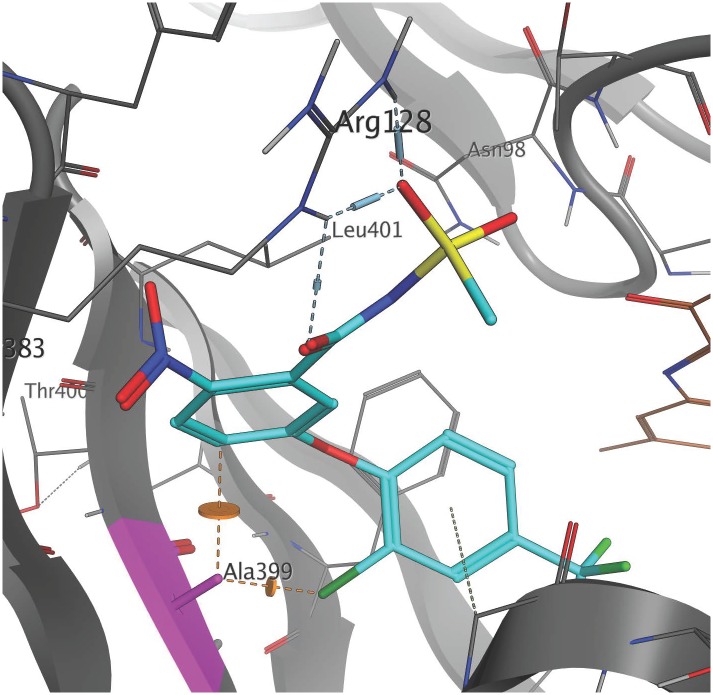
Position of the G399A mutation relative to the predicted binding mode of fomesafen. Fomesafen (cyan) was modeled into the binding-site of a homology model of a PPO2 protein sequence with G399A (gray) mutation. The alanine mutation is shown in pink. The introduction of G399A creates a couple of close steric contacts with the ligand (dashed orange lines and disks). The FAD cofactor (brown) is partially visible to the right. Several amino acid side chains are not shown, to improve clarity. The image was generated with MOE (*Molecular Operating Environment*, Chemical Computing Group ULC, Montreal, QC, Canada, H3A 2R7, 2017).

The high resolution X-ray crystal structure of *N. tabacum* PPO2 generated by Koch et al. (2004) provides three-dimensional protein structural information that is useful for understanding the role of critical amino acid residues in herbicide binding to PPO2 in plants. Here, as a first step toward rationalizing the impact of the G399A mutation on fomesafen binding, this protein structure (PDB entry 1SEZ) was used as a template to obtain structural models of the *A. palmeri* PPO2-fomesafen complex. In particular, the homology modeling workflow implemented in YASARA was used to derive models for both S-PPO2 and R-PPO2. The binding geometry of fomesafen in the binding-site of the S-PPO2 model was then predicted using in-house docking tools and transferred into the R-PPO2 model. The resulting visualization (Figure 2) suggests the following hypothesis for the molecular mechanism of G399A-induced fomesafen resistance in *A. palmeri* PPO2. The principal difference observed between the S-PPO2 and R-PPO2 models is the additional methyl (-CH_3_) group of A399 in R-PPO2 (highlighted pink). Although it comprises just a single heavy atom, this methyl group is oriented directly into a buried part of the PPO binding site, making the binding pocket smaller. This creates close steric contacts (indicated by orange lines in Figure 2), significantly below the sum of van-der-Waals radii, with the chloro-substituent of the terminal 2-chloro-4-trifluoromethylphenyl ring and the 2-position of the central phenyl ring. These repulsive interactions could reduce the magnitude of the protein-ligand binding energy and therefore weaken the inhibition of the PPO2 enzyme by fomesafen.

### Prevalence of G399A Mutation Among the Other Fomesafen-Resistant Populations

A PCR-based assay was designed to determine the presence of G399A mutation in 35 fomesafen-resistant field populations that we characterized in a previous study (Salas-Perez et al., 2017). We surveyed the presence of the G399A mutation among 130 R plants representing these 35 populations using cleaved amplified polymorphic sequences (CAPS) assay (Supplementary Figure S3). Collectively, about 16% (21 of 130) of R plants carried the G399A mutation. Further, only 14% of field populations (5 of 35) had R plants with this mutation and only two resistant populations had R plants predominantly carrying the G399 mutation. Our previous study on these same populations revealed that 26 of 35 populations harbor the ΔG210 mutation, and that the ΔG210 was present in 49% of all R plants tested (Salas-Perez et al., 2017).

### Fomesafen Dose Response Assay to Determine the Level of Resistance

**FIGURE 3:**
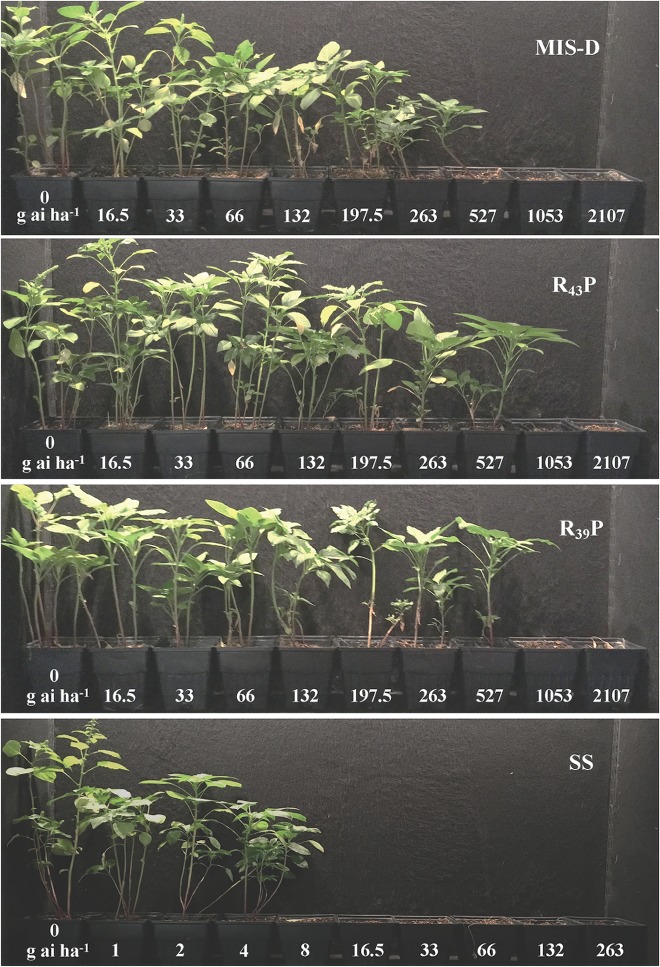
PPO-resistant and PPO-susceptible Palmer amaranth populations in a greenhouse dose-response experiment. Resistant (MIS-D, R_39_P, and R_43_P) and susceptible (SS) *A*. *palmeri* plants after 21 days of being sprayed with 9 doses of fomesafen. The first pot to the left of each photo was non-treated; fomesafen doses were in g ai ha^-1^.

**FIGURE 4:**
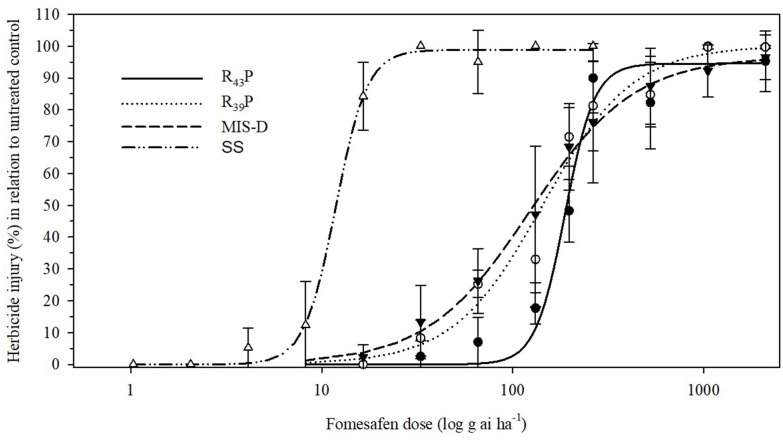
Visible injury (%) of Palmer amaranth SS (wild type), MIS-D (field population), and F1 populations (R_39_P and R_43_P) in response to nine fomesafen doses. Symbols and lines represent actual and predicted herbicide injury responses, respectively. Vertical bars represent ± standard errors of the mean. Data were fitted to a non-linear, log-logistic regression function, Y = d/[1+exp{b[log(x) – log(ED50)]}].

To determine the level of resistance to fomesafen in F1 population, we conducted the dose response assay with R_39_P and R_43_P F1 populations together with the original field population, MIS-D and SS. None of the resistant populations were killed 100% with 2X dose (528 g ai ha^-1^) of fomesafen. The SS population was controlled 100% with 8 g ai ha^-1^ (1/32X) (Figure 3). The amplitude of injury ratings among survivors of the 1X dose was high, ranging from total absence of symptoms to severe leaf necrosis and stunting. The estimated fomesafen dose that would cause 50% injury (ED_50_) of MIS-D, R_43_P, and R_39_P ranged from 123 to 187 g ai ha^-1^, whereas the ED_50_ for SS was 12 g ai ha^-1^. Thus, the levels of resistance to fomesafen in MIS-D, R_39_P, and R_43_P were 11-, 12-, and 16-fold, respectively, relative to the SS population (Figure 4 and Table 1). The G399A mutation confers dominant resistance to fomesafen as plants from R_43_P and R_39_P (derived from heterozygous male parent) manifests higher resistance, when compared to S plants (Figure 4). Also, the recommended dose of fomesafen (1X) did not control plants containing the G399 mutant allele either in homozygous or heterozygous state in screening survivors from MIS-D population (data not presented).

**Table 1:** Resistance levels of protoporphyrinogen oxidase-resistant *Amaranthus palmeri* populations.

Population	Biotype	ED_50_ (g ai ha^-1^)^a^	R/S^b^
MIS-D	PPO-resistant	124 (101–147)	11
R_39_P	PPO-resistant	143 (122–165)	12
R_43_P	PPO-resistant	187 (174–202)	16
SS	PPO-sensitive	12 (10–13)	


*^*a*^ED_*50*_, estimated dose of fomesafen herbicide required to cause 50% injury. Values in parenthesis are 95% confidence intervals. The 1X dose of fomesafen is 263 g ai ha^-*1*^.*

*^*b*^R/S, ED_*50*_ of the PPO-resistant biotype divided by the ED_*50*_ of the sensitive biotype.*

### G399A Mutant PPO Confers Broad Resistance to PPO-Inhibiting Herbicides

We performed a comprehensive *In vitro* enzyme activity assay of the wild type and recombinant PPO2 proteins with known resistance-conferring mutations ΔG210, R128L, and the novel mutation G339A using *A. tuberculatus PPO2* gene. Constructs were made to generate recombinant protein with ΔG210, R128L, and G339A mutations; other possible exchanges at G399 position; and wild type PPO2. Four G399 protein variants (G399D, G399V, G399C, and G399R) were soluble, but lacked enzyme activity; no enzyme activity was detected even after adding large amounts of enzyme to the assay mix. G399S had negligible activity, necessitating the addition of a high amount of enzyme to the assay mix before any measurable activity was detected. Among these recombinant variants, only G399A was active. Compared to ΔG210 and R128L, G399A had a very low activity (Supplementary Table S1). Resistance factors (IC_50_ values) were calculated after subjecting the recombinant protein to increasing concentrations of diphenylether, pyrimidinedione, triazolinone, N-phenyl-phthalimide, phenylpyrazole, and thiadiazole herbicides (Table 2). The G399A substitution had similar target resistance potency as ΔG210. The G399A and ΔG210 mutants exhibited higher IC_50_ than the wild type supporting that G399A mutation confers resistance to PPO-inhibiting herbicides. The R128L mutation seemed to endow resistance to diphenylethers, N-phenyl-phthalimides, pyrimidionedione, and triazolinones, but not to the other PPO herbicides tested. Based on the IC_50_ values, the G399A substitution afforded greater resistance level to most diphenylethers relative to ΔG210 or R128L.

**Table 2:** Effects of protoporphyrinogen oxidase (PPO) inhibitors on *in vitro* enzyme activity of recombinant *Amaranthus tuberculatus* PPO2 wild-type enzyme and resistant enzyme variants.

PPO chemical family	Herbicides	Wild type sensitivity [IC_50_] (M)	Resistance factor		
			
			G399A	ΔG210	R128L
Diphenyl ether	Acifluorfen	1.85 × 10^-8^	>541	>541	>541
	Fomesafen	1.32 × 10^-9^	>7576	12315	1479
	Lactofen	5.60 × 10^-11^	28167	18271	2812
	Oxyfluorfen	3.42 × 10^-11^	18548	982	74
Pyrimidinedione	Saflufenacil	1.10 × 10^-9^	570	1455	202
	Butafenacil	8.96 × 10^-12^	741	17578	23
Triazolinone	Carfentrazone	1.96 × 10^-10^	116	3957	3
	Sulfentrazone	1.75 × 10^-^8	282	571	571
N-phenylphthalimide	Flumioxazin	3.90 × 10^-11^	868	3157	2631
Phenylpyrazole	Pyraflufen-ethyl	5.19 × 10^-11^	11816	1083	1
Thiadiazole	Fluthiacet-methyl	6.69 × 10^-10^	3567	94	4
Oxadiazole	Oxadiazon	7.00 × 10^-10^	285	151	1
Other	Pyraclonil	1.39 × 10^-9^	799	782	0


### *A. palmeri* With G399A Mutation Displayed Cross-Resistance to Other Foliar-Applied PPO Herbicides

The field population, MIS-D, was resistant to the 1X dose of carfentrazone, flumioxazin, fomesafen, lactofen, fluthiacet-methyl, and pyraflufen-ethyl. The 1X dose of acifluorfen and saflufenacil caused >90% mortality in MIS-D while the other PPO-inhibiting herbicides caused 31–80% mortality (Salas-Perez et al., 2017). The number of plants resistant to the 2X dose of acifluorfen, carfentrazone, flumioxazin, lactofen, and saflufenacil in R_39_P was greater than the number of survivors at the 1X dose in the field population, MIS-D (Table 3), reflecting increased frequency (and level) of resistant plants in the F1 population. The frequency of resistant plants in R_39_P at the 2X dose was ≥40% with carfentrazone, flumioxazin, and lactofen. Although the survival rate was <20% with acifluorfen, fomesafen, and saflufenacil, the 2X dose of these herbicides did not kill all plants. This indicates that increasing the herbicide dose to 2X does not achieve complete control of this population. Besides, farmers cannot use 2X dose in the field because it is off-label and would increase the residual activity of the herbicide, which could be detrimental to the following (sensitive) crop in a crop rotation system. Cross-resistance to various families of PPO-inhibiting herbicides suggests that G399A mutation endows broad resistance to PPO-inhibiting herbicides.

**Table 3:** Response of PPO-resistant populations to the various foliar-applied protoporphyrinogen oxidase herbicides.

Protoporphyrinogen oxidase herbicides	Frequency of resistant plants (%)	
	
	MIS-D^a^	R_39_P^b^
Acifluorfen	1	8
Carfentrazone	20	64
Flumioxazin	40	60
Fomesafen	42	12
Lactofen	23	40
Saflufenacil	0	12


*^*a*^MIS-D field population was treated with the recommended dose of PPO-inhibiting herbicides.*

*^*b*^R_*39*_P purified population was treated with twice the recommended dose of PPO-inhibiting herbicides.*

## Discussion

Hundreds of weed species have evolved resistance to all classes of commonly used herbicides (Heap, 2018). However, there are relatively few cases of field-evolved resistance to PPO-inhibiting herbicides, despite its long history of widespread use. In weeds, the slow rate of evolution of resistance to PPO inhibitors mirrors that of resistance evolution to glyphosate, which took 22 years before detection (Duke and Powles, 2008; Heap, 2018). This situation reflects the high conservation of catalytic domains of these herbicide targets as mutations in these catalytic regions are generally lethal or physiologically detrimental. As both PPO isozymes are targets of PPO herbicides, redundancy in herbicide-sensitivity at the plant level may account for comparable infrequency of selection of resistant individuals in the field. *Amaranthus* species are among the first few weeds to evolve resistance against PPO-inhibiting herbicides (Patzoldt et al., 2006; Salas et al., 2016). Although both PPO1 and PPO2 are molecular targets of PPO-inhibitors, only PPO2 is found to be involved in target-site-based resistance in weeds. The preference of PPO2 as the resistance trait carrier is speculated to be an important aspect of evolution of resistance mechanism to PPO-inhibiting herbicides (Patzoldt et al., 2006; Salas et al., 2016; Giacomini et al., 2017; Salas-Perez et al., 2017; Dayan et al., 2018). A plausible reason for PPO2 as the preferred target for evolution may ensue from its distribution in target organelles. In *Amaranthus* species, PPO2 is dual-targeted to mitochondria and chloroplast. This dual-targeting of PPO2 circumvents the requirement of simultaneous selection of PPO1 mutation to endow resistance at the plant level. In other words, if PPO2 is not dual-targeted then mutation in PPO2 will only protect PPO activity in the mitochondria, not in the chloroplast; thus, the plant will still be sensitive to PPO inhibitors. Plants with dual-targeting of PPO2 will retain PPO activity in both compartments in the presence of PPO inhibitor, once a resistance-conferring PPO2 mutation is selected. The G399A mutation identified in *PPO2* gene in fomesafen-resistant plants may also occur in PPO1 as this site is also conserved across PPO isozymes in various species (Supplementary Figure S2); however, the resistance-conferring mutation has been selected only in PPO2 of *A*. *palmeri.* This affirms the significance of PPO2 in the evolution of resistance against PPO-inhibitors.

Glycine is a highly conserved amino acid residue in the evolution of proteins for its unique structural flexibility; thus, it plays different roles in the three-dimensional architectures of folded proteins (Branden and Tooze, 1999). The glycine at 399 position in the PPO2 of *A*. *palmeri* is also highly conserved and is a part of the catalytic domain, suggesting that it is essential for protein functionality. A mutation at this site is predicted to cause a change in stereochemistry and could result in the conformational change of the PPO2 protein during folding. The structural difference between the wild type and mutated (G399A) PPO2 variant from *A*. *palmeri* with fomesafen was validated by homology modeling and computational docking (Figure 2). The computational analysis showed that the steric hindrance caused by the additional methyl group of alanine substitution at 399 position in PPO2 changes the conformation of the binding pocket, making it less accessible for herbicide binding. The resulting hypothesis for the molecular mechanism of resistance owing to the G399A mutation is that the steric hindrance caused by the additional methyl group with alanine at the 399 position causes a small, but critical, change in the shape of the binding pocket, making it less accessible for herbicide binding.

The G399A mutation is less prevalent in the field than the other known mutations among PPO-resistant *A. palmeri*. The absence of other exchanges at this position in MIS-D population was supported by *in vitro* data where only the G399A recombinant protein variant was active out of six possible amino acid exchanges. Perhaps only the G399A exchange can be physiologically tolerated, and any other mutations at this site are lethal or too detrimental biochemically, such that plants harboring alternate mutations at G399 do not exist. Further, the low frequency of resistant plants carrying G399A may also be indicative of a fitness cost of this mutation, which is evident from a very low enzyme activity (approximately 3%) with G399A mutation *in vitro* experiment. Thus, evaluation of fitness penalties associated with the new G399A mutation in the presence and absence of selection would be informative for the development of better control strategies for management of resistance to PPO-inhibiting herbicides. Nevertheless, the continued use of the same PPO inhibitor favors the R plants and would perpetuate the resistant alleles. One more cycle of purification in our experiment increased the resistance level of the next generation.

*In vitro* data with G399A, ΔG210, and R128L PPO2 variant showed different degrees of cross-resistance among and between the different classes of PPO inhibitors due to the difference in chemical structure that influences the binding and orientation of the inhibitor in the enzyme catalytic site. PPO herbicides compete with the substrate, protoporphyrinogen IX, of PPO2 enzyme by mimicking parts of the substrate. Different inhibitors have different mimicking modes depending on its structure. Herbicides belonging to the same chemical family have the same base structures and usually behave similarly in the way they interact with the enzyme. It is apparent that diphenylethers are particularly highly affected by G399A, ΔG210, and R128L mutations. This is probably because diphenylethers were primary selectors for the evolution of PPO-inhibitor-resistant weeds.

Both whole plant bioassay and *in vitro* assay revealed that G399A mutation endowed broad cross-resistance to diphenylether, pyrimidinedione, triazolinone, N-phenylphthalimide, phenylpyrazole, thiadiazole, and oxadiazole PPO herbicide families. The position of G399 is critical to the herbicide-binding site, thus the replacement of G399 with alanine weakens the affinity of PPO2 enzyme to different PPO inhibitors. According to the protein structural models derived here, the methyl group of alanine protrudes into the buried part of the PPO-binding site causing the pocket to constrict. Thus, some of the ring structures and side chains of amino acids and those of various PPO-inhibiting herbicides would clash. The drop in frequency of fomesafen-resistant plants in the F1 population relative to the original field population is because of the double dose used on the F1 population (Table 3). The resistance level of R_39_P to fomesafen (12-fold) turned out to be similar to that of the original population, MIS-D (11-fold) (Table 1). The 1X dose is 263 g ai ha^-1^. Fifty percent of R_39_P could be killed with less than the 1X dose (143 g ai ha^-1^) of fomesafen. Therefore, treating the F1 population with 2X dose of fomesafen reduced the number of survivors compared to the original population, which was treated with only 1X dose (Table 3). Except for fomesafen, resistance to representatives of the other groups of PPO herbicides increased in the F1 population, as expected.

The cross-resistance pattern to various PPO herbicide families and the resistance levels are complex. There is indication that besides zygosity and level of purification of a population, non-target genes contribute to PPO resistance in some populations, including MIS-D. All the 5 R lines we used for sequencing carried the G399A mutation. Also, all survivors from MIS-D population contained only this mutation, but these plants exhibit different levels of resistance in the screening experiments (data not shown). Non-target site mechanisms involve many genes and can co-exist with target-site mutation. This can modify the resistance level and makes the inheritance pattern messy. Follow-up research is needed to sort out the involvement of non-target-site-resistance mechanisms in the evolution of Palmer amaranth resistance to PPO inhibitors.

The ΔG210 also caused broad resistance to PPO inhibitors although G210 is not located in the catalytic site. Previous studies showed that the G210 plays a key role in stabilizing the α-8 helix of PPO2. Deletion of G210 unravels the last turn of the helix and results in about 50% enlargement of the active site cavity (Dayan et al., 2010), thereby allowing both the substrate and PPO inhibitors to penetrate into the binding pocket. The ΔG210 does not affect the affinity to protoporphyrinogen IX, but reduces the affinity of inhibitor for PPO2 (Dayan et al., 2010), thus rendering the PPO inhibitor less or not effective.

The R128L mutation caused resistance to diphenylethers, N-phenylphthalimides, pyrimidionedione, and triazolinones, but not to phenylpyrazole, thiadiazole, oxadiazole, and pyraclonil *in vitro* assays. R128 is in the pocket between the active site and the entryway of the substrate, and is crucial for stabilizing the substrate in the catalytic domain (Heinemann et al., 2007; Hao et al., 2013, 2014; Dayan et al., 2018). The mutation of arginine, a charged amino acid that is often involved in salt bridges, to a hydrophobic leucine causes a drastic change in the enzyme conformation. Computational modeling by Hao et al. (2014) revealed that replacement of R128 with hydrophobic glycine removed important hydrogen-bonding interactions with acifluorfen, fomesafen, and sulfentrazone, resulting in reduced binding of these inhibitors. Oxadiazole, on the other hand, does not form hydrogen bond with R128, thus mutation at this position does not affect its binding to the enzyme and the plant remains sensitive to oxadiazoles.

With this recent discovery of G399A mutation in PPO-inhibitor-resistant *A. palmeri*, it seems that the *PPO2* gene of *A. palmeri* is more prone to resistance selection than the *PPO2* gene of *A. tuberculatus*. The evolution of G399A mutation alongside ΔG210 and R128 mutations illustrates the broader adaptability of *A. palmeri* to herbicide selection pressure than other weed species. Previously, we found that R plants carrying different PPO2 mutations can co-exist in one field and that some R plants do not carry any target site mutations (Salas-Perez et al., 2017). This indicates involvement of non-target-site-resistance mechanisms in some populations in addition to the presence of target site mutations, which could increase the field-level resistance.

## Conclusion

The novel mutation G399A confers broad cross-resistance to PPO-inhibiting herbicides. This mutation is relatively rare, detected in only 16% of R plants and is predominant in only 2 of 35 PPO-resistant *A. palmeri* populations in Arkansas. This mutation has not been reported to occur in other PPO-inhibitor-resistant species. The G399A mutation is notable compared to the other known mutations in that it inhibits herbicide binding through simple steric hindrance. The G399A was not anticipated in conferring resistance to PPO inhibitors in spite of it being in the catalytic domain, maybe because it is a simple substitution. However, in nature, G399A has been selected to confer resistance against PPO-inhibiting herbicides. Multiple PPO2 mutations can accumulate in one plant in multiple alleles, most likely via gene flow. Whether the enzyme can tolerate multiple mutations in one allele and whether multiple resistance-conferring PPO mutations in one plant would result in even higher resistance levels are yet to be determined.

## Accession Numbers

PPO2 sequence data from this article can be found in GenBank (http://www.ncbi.nlm.nih.gov) under the following accession numbers: MK408971, MK408972, MK408973, MK408974, MK408975, MK408976, MK408977, and MK408978.

## Author Contributions

GR participated in the design of molecular biology experiments and coordination, performed the molecular experiments, analyzed the data, and wrote the manuscript. RS-P, RA, and MK designed and performed *in vitro* assays and analyzed the data. IC and TM performed computational modeling of the novel mutation. RS-P participated in field sampling of Palmer amaranth populations with NR-B, conducted the resistance bioassays, produced the F1 populations, performed the herbicide dose-response assays, and participated in the manuscript preparation. AL and MN performed the herbicide assays and helped in genotyping. RA conceived the enzyme activity assay study and helped in coordination overall. AP provided the technical input in addressing comments from reviewers, pertaining to mutant PPO enzyme activity, providing details on methodology and supplemental data. NR-B conceived the whole Palmer amaranth adaptation project, obtained funding for the project, organized and led the collection of Palmer amaranth samples and field histories, conceptualized the phenotyping and molecular biology experiments, directed the research implementation, and participated in the manuscript preparation and revisions.

## Conflict of Interest Statement

RA, MK, IC, TM, and AP were employed by BASF Company. The remaining authors declare that the research was conducted in the absence of any commercial or financial relationships that could be construed as a potential conflict of interest.
