# Supplementary material for: A Novel Single-Site Mutation in the Catalytic Domain of Protoporphyrinogen Oxidase IX (PPO) Confers Resistance to PPO-Inhibiting Herbicides
Source: Front Plant Sci. 2019 May 15;10:568. doi: 10.3389/fpls.2019.00568 (PMC6530635; doi:10.3389/fpls.2019.00568)
Supplement: Supplementary file 1 [file Data_Sheet_1.PDF]

Supplementary Materials and Methods

Recombinant expression, purification and in vitro inhibition studies of PPO2

Preparation of auto-induction medium
Single colonies were picked and used to inoculate 1.5 ml of LB or auto-induction media (AIM) containing 50 µg/ml kanamycin. AIM contains the following components: 1% N-Z amine AS (Gibco), 0.5% yeast extract (Gibco), 25 mM Na2HPO4 (Merck), 25 mM KH2PO4 (Merck), 50 mM NH4Cl (Merck), 5 mM Na2SO4 (Merck), 2 mM MgSO4 (Merck), 0.5% glycerol (Gerbu), 0.05% glucose (Sigma) and 5.6 mM α-lactose (Sigma). In addition, 0.2x or 1x trace metals were added from a 1000x stock solution containing 0.1 M FeCl3, 1M CaCl2, 1M MnCl2-4H2O, 1 M ZnSO4-7H2O, 0.2 M CoCl2-6H2O, 0.1 M CuCl2-2H2O, 0.2 M NiCl2-6H2O, 0.1 M Na2MoO4-2H2O, and 0.1 M H3BO3. LB and TB cultures were induced with 0.1 mM IPTG when the OD600 of at least three cultures reached 0.6 – 0.7.

Preparation of substrate
8.1 mg protoporphyrin - IX [CAS-No. 553-12-8] were weighed into a 100 ml three-necked flask. Then 33.5 ml of the solvent (10mM KOH, 20% Ethanol in deionized water) was added and stirred for 30 min in the light. 40.0 g of sodium alum (4-5% Na) [CAS-No. 11110-52-4] from ABCR (AB 109084) were placed in a 100 ml three-necked flask. Then, the protoporphyrin solution was added dropwise. All steps above were performed under nitrogen. Reduction took place for 10min. After reduction the protoporphyrin was filtered in another flask also filled with sodium amalgam. Wash buffer (33.5 ml) is first reduced in the same flask where the protoporphyrin was reduced, then also filtered and added to the flask with protoporphyrin (Wash buffer was prepared using 250mM Tris HCl PH 8.5, 5mM EDTA, 5mM GSH in deionized water). Another reduction took place for 10 minutes. Then the substrate was filtered into flask under nitrogen and immediately aliquoted in 1.5ml eppendorf tubes (800 ml aliquots). 400µl Paraffin was added, and the tubes frozen on dry ice and stored dark at -80°C. The final concentration of the substrate was 216 µM.

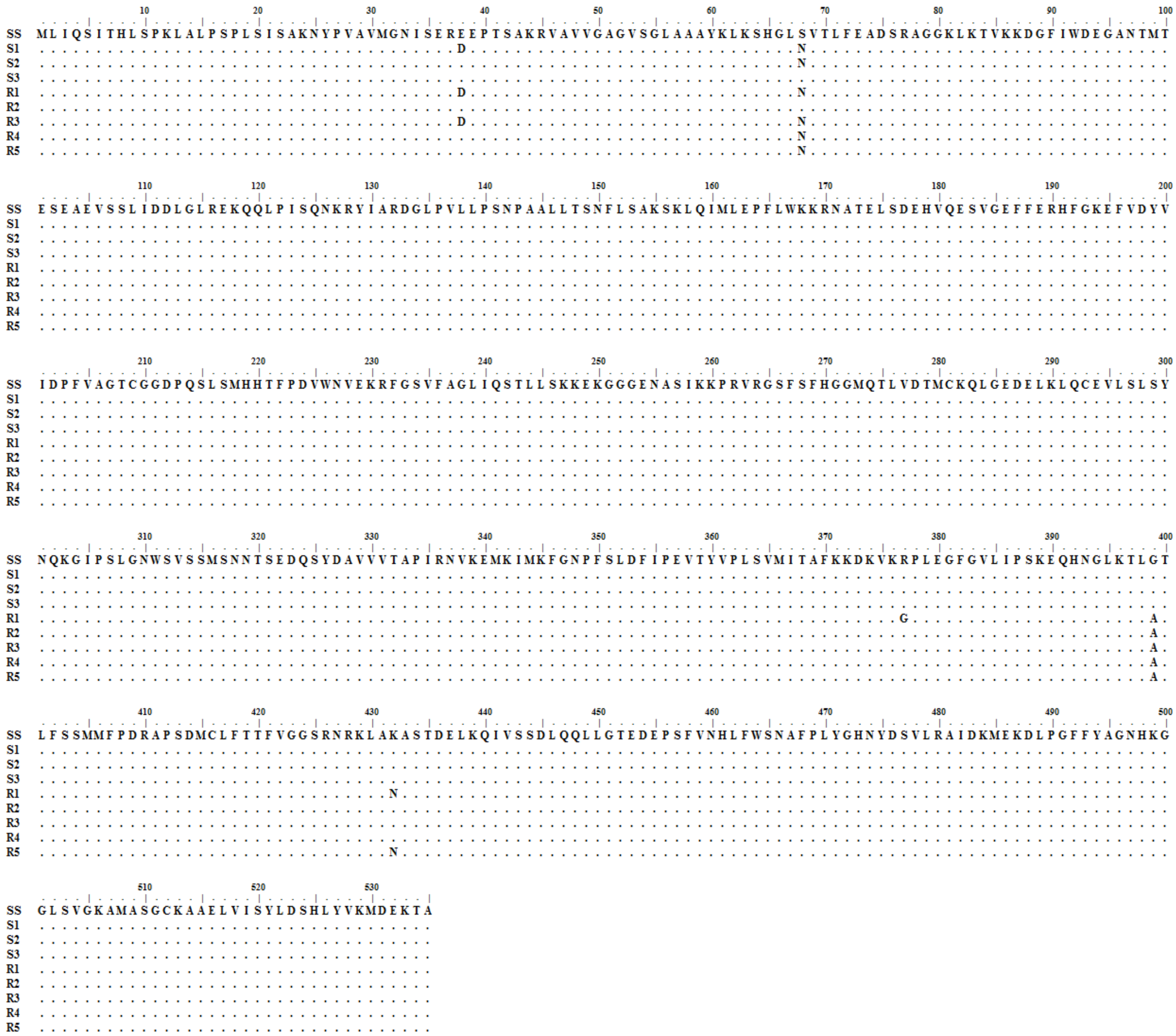

Fig. S1: PPO2 amino acid alignments

Full-length amino acid sequence of R1-R5 and S1-S3 from the same population was aligned with *A. palmeri* SS (ATE88443) as a reference. Dots represent identity at that position compare to SS. Amino acid numbers are indicated above the reference sequence.

399

Amaranthus palmeri (ATE88443.1)	PSKEQ-HNGLKTLGTLFSSMMFPDRAPSDMCLFTTFV
Amaranthus hypochondriacus (ABS72165.1)	PSKEQ-HNGLKTLGTLFSSMMFPDRAPSDMCLFTTFV
Amaranthus tuberculatus (ABD52326.1)	PSKEQ-HNGLKTLGTLFSSMMFPDRAPSDMCLFTTFV
Spinacia oleracea (XP_021854936.1)	PSNEQ-HNGLKTLGTLFSSMMFPDRAPSDVYLYTTFV
Chenopodium quinoa (XP_021775105.1)	PSNEQ-HNGLKTLGTLFSSMMFPDRAPSDVYLYTTFV
Beta vulgaris(XP_010693290.1)	PSNEQ-HNGLKTLGTLFSSMMFPDRAFSDMYLYTTFV
Vitis vinifera (XP_002263321.1)	PSKEQ-QNGLKTLGTLFSSMMFPDRAPNDLYLYTTFI
Ziziphus jujube (XP_015867153.1)	PSKEQ-NNGLKTLGTLFSSMMFPDRAPNDLYLYTTFV
Jatropha curcas (XP_020534796.1)	PSKEQ-QNGLKTLGTLFSSMMFPDRAPDDLYLYTTFI
Prunus avium (XP_021833288.1)	PSKEQ-KNGLKTLGTLFSSMMFPDRAPSDLYLYTTFV
Malus domestica (XP_008341682.1)	PSKEQ-KNGLKTLGTLFSSMMFPDRAPSDLHLYTTFV
Theobroma cacao (EOY02725.1)	PSKEQ-QNGLKTLGTLFSSIMFPDRAPNNLYLYTTFV
Juglans regia (XP_018849245.1)	PSKEQ-QNGLKTLGTLFSSMMFPDRAPNDLYLYTTFV
Carica papaya (XP_021890416.1)	PSKEQ-QNGLKTLGTLFSSVMFPDRAPSDLHLYTTFV
Herrania umbratica (XP_021299062.1)	PSKEQ-QNGLKTLGTLFSSIMFPDRAPNNLYLYTTFV
Momordica charantia (XP_022133942.1)	PSVEQ-RNGLRTLGTLFSSMMFPDRALSDEYLYTTFI
Gossypium raimondii (KJB76437.1)	PSKEQ-QNGLKTLGTLFSSVMFPDRAPNNLYLYTTFV
Cucumis sativus (XP_011656375.1)	PSSEQ-QNGLRTLGTLFSSMMFPNRASNDEYLYTTFI
Cajanus cajan (XP_020218703.1)	PSKEQ-QNGLKTLGTLFSSMMFPDRAPSDLYLYTTFI
Cucumis melo (XP_008441360.1)	PSLEQ-QNGLRTLGTLFSSMMFPNRASNDEYLYTTFI
Gossypium hirsutum (XP_016747921.1)	PSKEQ-QNGLKTLGTLFSSVMFPDRAPNNLYLYTTFV
Nicotiana tabacum (XP_016496870.1)	PSKEQ-QHGLKTLGTLFSSMMFPDRAPNNVYLYTTFV
Gossypium raimondii (KJB76435.1)	PSKEQ-QNGLKTLGTLFSSVMFPDRAPNNLYLYTTFV
Capsicum annuum (XP_016570686.1)	PSKEQ-KHGLKTLGTLFSSMMFPDRAPSNVYLYTTFV
Cajanus cajan (KYP63379.1)	PSKEQ-QNGLKTLGTLFSSMMFPDRAPSDLYLYTTFI
Solanum lycopersicum (NP_001335305.1)	PSQEQ-KHGLKTLGTLFSSMMFPDRAPNNVYLYTTFV
Nicotiana tabacum (XP_016434987.1)	PSKEQ-QHGLKTLGTLFSCMMFPDRAPNNVYLYTTFV
Arachis duranensis (XP_015971359.1)	PSKEQ-QNGLKTLGTLFSSMMFPDRAPSDLHLYTTFV
Solanum pennellii (XP_015070810.1)	PSQEQ-KHGLKTLGTLFSSMMFPDRAPNNVYLYTTFV
Lupinus angustifolius (XP_019458023.1)	PSKEQ-QNGFKTLGTLFSSMMFPDRAPSDLHLYTTFV
Hevea brasiliensis (XP_021645933.1)	PSKEQ-ENGLKTLGTLFSSMMFPDRAPSDLYLYTTFV
Solanum tuberosum (XP_006356026.1)	PSEEQ-KHGLKTLGTLFSSMMFPDRAPNNVYLYTTFV
Brassica napus (XP_009121685.1)	PSIEQ-KHGFKTLGTLFSSMMFPDRCPSDLHLYTTFI
Coffea canephora (CDP16674.1)	PSKEQ-QNGLKTLGTLFSSMMFPDRAPNDTYLYTTFV

Brassica napus (XP_013667641.1)	PTKEQ-KRGFKTLGTLFSSMMFPDRCPSDLHLYTTFI
Eucalyptus grandis (XP_010034660.1)	PSAEQ-KNGLRTLGTLFSSMMFPDRAPTELYLYTTFI
Nelumbo nucifera (XP_010250782.1)	PSKEQ-QNGLRTLGTLFSSMMFPDRAPGDLYLYTTFI
Brassica napus (CDY57892.1)	PTKEQ-KHGFKTLGTLFSSMMFPDRCPSDLHLYTTFI
Oryza sativa (XP_025880545.1)	PYSKEQHNGLKTLGTLFSSMMFPDRAPSDMCLFTTFV
Setaria italic (XP_004976030.1)	PYKEQQKHGLKTLGTLFSSMMFPDRAPDDQYLYTTFV
Panicum hallii (XP_025825969.1)	PYKEQQKHGLKTLGTLFSSMMFPDRAPDDQYLYTTFV
Sorghum bicolor (XP_002446710.1)	PYKEQQKHGLKTLGTLFSSMMFPDRAPDDQYLYTTFV
B. distachyon (XP_024311695.1)	PYKEQQKYGLKTLGTLFSSMMFPDRAPNDQHLFTTFV
Zea mays (NP_001105004.2)	PYKEQQKHGLKTLGTLFSSMMFPDRAPDDQYLYTTFV
Ananas comosus (XP_020111709.1)	PSKEQE-NGLKTLGTLFSSMMFPDRAPSDQYLYTTFL
Triticum aestivum (SPT18865.1)	PFKEQQKHGLKTLGTLFSSMMFPDRAPNDQYLFTTFI
Elaeis guineensis (XP_019708490.1)	PFKEQQKHGLKTLGTLFSSMMFPDRAPNDQYLYTTFV

Arabidopsis thaliana (NP_196926.2)	GQLHPRSQGVETLGTIYSSSLFPGRAPPGRTLILNYI
Chenopodium quinoa (XP_021730637.1)	GQLHPRSQGVETLGSIYSSSLFPGRAPPGRTLILSYI
Beta vulgaris (XP_010693036.1)	GQLHPRSQGVETLGTIYSSSLFPGRAPPGRILILSYI
Amaranthus tuberculatus (ABD52324.1)	GQLHPRSQGVETLGTIYSSSLFPGRAPPGRTLILSYI
Vitis vinifera (CAN69262.1)	GQLHPRSQGVETLGTIYSSSLFPNRAPPGRILLLNYI
Citrus clementina (XP_006444625.1)	GQLHPRSQGVETLGTIYSSSLFPNRAPAGRVLLLNYI
Populus euphratica (XP_011025274.1)	GQLHPRSQGVETLGTIYSSSLFPNRAPTGRILLLNYI
Populus trichocarpa (XP_002320252.2)	GQLHPRSQGVETLGTIYSSSLFPNRAPAGRILLLNYI
Medicago truncatula (XP_013469143.1)	GQLHPRSQGVQTLGTIYSSSLFPNRAPPGRVLLLNYI
Gossypium hirsutum (XP_016695806.1)	GQLHPRSQGIETLGTIYSSSLFPNRAPSGRVLLLNYI
Brassica napus (XP_013666368.1)	GQLHPRTQKVETLGTIYSSSLFPNRAPPGRVLLLNYI
Carica papaya (XP_021897188.1)	GQLHPRSQGVETLGTIYSSSLFPNRAPAGRVLLLNYI
Gossypium hirsutum (XP_016710428.1)	GQLHPRSQGIETLGTIYSSSLFPNRAPSGRVLLLNYI
Nelumbo nucifera (XP_010240808.1)	GQLHPRSQGVETLGTIYSSSLFPNRAPPGRVLLLNYI
Nicotiana tabacum (NP_001313110.1)	GQLHPRTQGVETLGTIYSSSLFPNRAPKGRVLLLNYI
Arabidopsis lyrata (XP_020877341.1)	GQLHPRTQGVETLGTIYSSSLFPNRAPPGRILLLNYI
Momordica charantia (XP_022146407.1)	GQLHPRSQGVTTLGTIYSSSLFPNRAPDGRVLLLNYI
Camelina sativa (XP_010428671.1)	GQLHPRTQGVETLGTIYSSSLFPNRAPPGRVLLLNYI
Glycine max (XP_003536005.1)	GQLHPRSQGVETLGTIYSSSLFPNRAPPGRVLLLNYI
Solanum lycopersicum (NP_001335308.1)	GQLHPRSQGVETLGTIYSSSLFPNRAPNGRVLLLNYI

Solanum pennellii (XP_015058045.1)	GQLHPRSQGVETLGTIYSSSLFPNRAPNGRVLLLNYI
Capsicum chinense (PHU30347.1)	GQLHPRSQGVETLGTIYSSSLFPNRAPNGRVLLLNYI
Capsicum baccatum (PHT59762.1)	GQLHPRSQGVETLGTIYSSSLFPNRAPNGRVLLLNYI
Oryza sativa (XP_015626054.1)	GQLHPRSQGVETLGTIYSSSLFPNRAPAGRVLLLNYI
Panicum miliaceum (RLM91355.1)	GQLHPRSQGVETLGTIYSSSLFPNRAPAGRVLLLNYI
Setaria italica (XP_004967639.1)	GQLHPRSQGVETLGTIYSSSLFPNRAPAGRVLLLNYI
Zea mays (NP_001105564.2)	GQLHPRSQGVETLGTIYSSSLFPNRAPDGRVLLLNYI
B. distachyon (XP_003567497.2)	GQLHPRSQGVETLGTIYSSSLFPNRAPAGRVLLLNYI
Sorghum bicolor (XP_002455484.1)	GQLHPRSQGVETLGTIYSSSLFPNRAPAGRVLLLNYI
Aegilops tauschii (XP_020151190.1)	GQLHPRSQGVETLGTIYSSSLFPNRAPAGRVLLLNYI
Phoenix dactylifera (XP_008800315.1)	GQLHPRSQGVETLGTIYSSSLFPNRAPPGRVLLLNYI
Ananas comosus (OAY70844.1)	GQLHPRSQGVETLGTIYSSSLFPNRAPPGRVLLLNYI

Fig. S2: Multiple sequence alignment of eukaryotic PPO proteins showing conservation of G399 at the protein level
Representative eukaryotic species aligned using Cobalt analysis (NCBI). Alignment with *A. palmeri* PPO2 is shown on the top line and the region around G399 position is used for comparison. Arrowheads represent 399 position in *A. palmeri* PPO2 protein. Dark purple and light purple box represents conserved glycine for, PPO2 and PPO1 protein, respectively across representative eukaryotic species

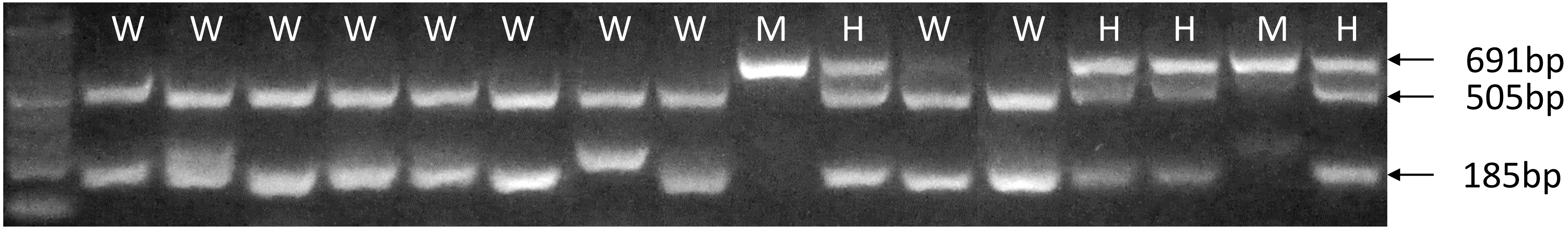

Fig. S3: Representation of detection of G399A mutation on 3 % agarose gel.
Genomic DNA isolated from fomesafen resistant plants was subjected to PCR using *PPO2* gene specific primers flanking mutation site. The resulted PCR product was digested with *Rsa*I restriction enzyme to distinguish the WT allele and different state of mutant allele. M refers to presence of mutant allele in homozygous state, H refers to presence of mutant allele in heterozygous state, W refers to wild type allele (absence of mutant allele)

Table S1: Relative activity of purified enzymes

Enzyme	Maximum fluorometric measurements(A) (FU/min)	Amount of each enzyme(B) (ng)	Enzyme activity C=A/B	% Relative activity (C/enzyme activity of WT*100)
Wild-type	1128	25	45	100
G339A	400	300	1.3	3
ΔG210	2172	200	11	24
R128L	1899	100	19	42
